# A Comparative Study of Apical Microleakage Using the Conventional Lateral Condensation and Mechanical Lateral Condensation Techniques

**Published:** 2008-07-10

**Authors:** Shahriar Shahriari, Seyed Mohsen Jalalzadeh, Reza Moradkhany, Hasan Abedi

**Affiliations:** 1 *Department of Endodontics, Dental School, Hamadan University of Medical Sciences, Hamadan, Iran*; 2 *General Practitioner, Hamadan, Iran*

**Keywords:** Dental Leakage, Endodontics, Instrumentation, Root Canal Preparation

## Abstract

**INTRODUCTION:** This study compared apical dye penetration using lateral condensation technique (LC) and LC technique with a reciprocal handpiece (mechanical lateral condensation or MLC) as a new method.

**MATERIALS AND METHODS:** Forty-eight human extracted straight canine teeth were used. After crown amputation, the teeth were randomly divided into four experimental groups of 10 teeth each and two negative and positive control groups of 4 teeth each. The groups were as follows: IA, 10 obturations completed by operator A using the LC technique; Group IB, 10 obturations completed by operator B using the LC technique; Group IIA, 10 obturations completed by operator A using the MLC technique; and Group IIB, 10 obturations completed by operator B using the MLC technique. All roots were placed in 2% methylene blue dye and centrifuged at 3000 rpm for 3 minutes. Following centrifugation, the roots were cut along their long axis and evaluated under a stereomicroscope to measure the depth of dye penetration.

**RESULTS:** A t-test showed that the teeth which were filled by the MLC technique had less dye penetration in comparison with LC technique (P<0.05).

**CONCLUSION:** This *in vitro *study illustrates that canals obturated with the MLC technique had superior apical seal than canals filled with the LC technique.

## INTRODUCTION

The goals of root canal treatment are to clean the root canal thoroughly, remove bacteria and debris, shape the canal and fill it with precision. Obturation provides a seal that prevents reinfection of the canal and subsequent leakage into the periradicular tissues (1). It is suggested that the most common cause of endodontic failures is incomplete obturation of the root canal (60%) (2). An important goal in filling the canal filling is to maximize the volume of root core material packed into the canal and to minimize the volume of root canal sealers (3). Currently, the two most popular gutta-percha obturation techniques are cold lateral condensation (LC) and warm vertical condensation (WVC) (4).

Lateral condensation is the obturation technique most widely taught in dental schools and used by practitioners, and is still the standard with which all other techniques are compared. Authors have investigated methods of partially combining the two techniques, hoping to combine the advantages of both (4,5). Gound *et al. *described mechanical lateral condensation (MLC), an alternative method of obturation that uses frictional heat to thermoplasticize gutta- percha in the canal. Mechanical lateral condensation involves placing a master cone in the canal, followed by a nickel-titanium spreader activated with a reciprocating-action handpiece (6). Several manufacturers, including NSK (Nakanishi Inc., Tokyo, Japan), Kerr (Kerr/ Sybron, Orange, CA, USA), and Myco (Myco Union Broach, York, PA, USA) have marketed reciprocating handpieces. Numerous studies have evaluated the apical sealing ability of root canal fillings using methods such as dye leakage (7). Dye penetration is commonly used to evaluate leakage due to its simplicity and cost- effectiveness (8). The purpose of this study was to compare the apical microleakage when the same type and size of gutta-percha cones were compacted into the canals using the MLC or traditional LC techniques as well as to assess the effect of the operator's skill on completely sealing the canals.

## MATERIALS AND METHODS

Forty-eight freshly extracted canine teeth with mature apices were selected. Preoperative radiographs were taken to confirm absence of root caries, fractures, multiple canals, calcifications, radicular resorption or excessive curvatures. After crown amputation, the obtained canals, 16-18 mm in length, were enlarged to a size 35 master apical file with a K-file (Mani, Tochigi, Japan) and flared to size #80 using step- back technique. With a size #10 K-file, apical patency was performed for all canals, after instrumentation the canals were irrigated with 17% EDTA and 5.25% NaOCl to remove the smear layer. Teeth were randomly divided into four experimental and two negative and positive control groups. In the four experimental groups, one-half of the obturations were accomplished using the LC technique and one-half using the MLC technique.

For the LC technique, a size #35 master cone (Aria Dent, Tehran, Iran) with AH26 sealer (DeTrey. Dentsply, Konstanz, Germany) was placed to the working length, then a medium-fine NiTi finger spreader (Hygenic Corp., Chicago, IL) was advanced into the canal until resistance occurred (9). The spreader was rotated in a reciprocating motion with apical pressure, attempting to penetrate to within 1 mm of the working length. After apical progression stopped, apical pressure was maintained for approximately 60s and then the spreader was removed (9). A size #20 gutta-percha accessory cone (Aria Dent, Tehran, Iran) was inserted into the space created in the canal and advanced apically as far as possible. The process was repeated until the spreader could not penetrate into the coronal one- third of the canal. The other one-half of the obturations were accomplished using the MLC technique. The same canals, spreaders, and materials that were used with the LC obturations were also used with the MLC technique.

The spreader was seated into the head of the reciprocating handpiece NSK (TEP-E10R, Nakanishi Inc., Tokyo, Japan). A rubber stopper was placed on the spreader at working length and then inserted into the canal alongside the master cone until resistance was felt. The handpiece was set at the maximum speed setting. The handpiece was activated and a light force was used to slowly advance the spreader apically to the desired or maximum level of penetration. Activation was continued at this level for 1-5 s and during removal of the spreader. Accessory cones were placed and obturation was completed using the same procedures that were used with traditional LC (6).

Operator A was a general dentist and did not have experience with MLC but had experience using LC. Operator B was endodontist experienced MLC and LC techniques. The following four experimental groups were created: Group IA, 10 obturations completed by operator A using the LC technique; Group IB, 10 obturations completed by operator B using the LC technique; Group IIA, 10 obturations completed by operator A using the MLC technique; and Group IIB, 10 obturations completed by operator B using the MLC technique.

In the positive control group, the canals were not obturated. In the negative control group, two canals were obturated with the MLC technique and two canals with the LC technique. In order to create coronal seal, 3 mm of the root canal obturation was removed and replaced with temporary filling material (Coltozol). After placing the samples in an incubator at 37**o**C for24 hours, the resected area and the surface of all roots in experimental and positive control groups were then covered with two layers of nail polish, except for the apical area. In the negative control group all surfaces of the roots, including the apical area, were covered with two layers of nail polish. The roots were then placed in 2% methylene blue dye and centrifuged at 3000 rpm for 3 minutes. Following centrifugation, the roots were cut along their long axis and evaluated under a stereomicroscope to measure the depth of dye penetration. The results were analyzed using t-test.

## RESULTS

The negative controls showed no dye penetration while, the positive controls showed completely dye penetration. Mean and standard deviation of leakage for experimental groups LCA, LCB, MLCA, and MLCB were 2.75±0.85, 3.05±1.87, 2.50±1.35, and 1.45±0.49 mm, respectively. The mean leakage for all MLC and LC obturations were 1.9 and 2.9 mm respectively. The difference was statistically significant (P<0.05).

The difference between the two operators in groups IA and IB was not statistically significant, but in groups IIA and IIB, the difference was statistically significant (P<0.05).

## DISCUSSION

Under the condition of this *in vitro *study, leakage in MLC group was significantly lower than LC group and experience with MLC significantly reduced dye leakage.

There are many methods to evaluate the sealing quality of filling materials (8), but dye penetration is most commonly used. This technique is simple to carry out but appears to overestimate microleakage ie, more than the bacteria infiltration method. This may be due to the difference between the sizes of molecular dyes and bacteria (10). In this study, a centrifugation method was used for determining microleakage in the samples (11). Another study (12) has showed that this method leads to increased dye penetration and therefore more meticulous than the conventional plunging method. In addition, this method requires less time for evaluation of dye penetration. For control of the method, two positive and negative control groups were used. Dye penetration in the positive control group and absence of dye penetration in the negative control group confirmed the integrity of dye penetration. In this study, we tried to create similar conditions for the samples. The same type and size of master cones, spreaders, and size of accessory points were used. In this study, dye penetration in teeth obturated with the LC technique was compared to the MLC techniques. The results showed that microleakage in the MLC technique was less than in the LC technique (P<0.05). Gound *et al. *compared the weight of gutta-percha between LC and MLC techniques. They found that the MLC obturations were significantly heavier than LC obturations (6). In another study the same group also compared the effect of spreader and accessory cone size on density of obturation using conventional or mechanical lateral condensation. They found that the MLC fills were significantly heavier and had greater depth of penetration than conventional lateral condensation. The best combination for heavy fills was MLC, fine-medium spreaders and fine accessory cones. The greatest mean accessory cone depth occurred with MLC, fine-medium spreaders and size 25 accessory cones (13). Jarrett *et al*. compared the apical density of several obturation techniques used in palatal roots of extracted maxillary molars. Results recommended that Thermafil, mechanical lateral and warm vertical (Schilder Technique) obturation techniques created more complete obturation at the 2 and 4 mm levels than cold lateral and warm vertical compaction (continuous wave) (14). Two different operators were employed to investigate the effect that different levels of experience may have on the results. The difference between two operators in groups IA and IB was not statistically significant, however in groups IIA and IIB the difference was significant. This result suggests that a difference in experience levels was important.

## CONCLUSION

We may conclude that it seems the MLC technique is a better and more suitable method for root canal obturation. However further studies are required to confirm this relationship as well as evaluating the amount of gutta-percha used and spreader depth penetration and so forth.
